# Negative Parenting Mediates the Longitudinal Association between Parental Internalizing Symptoms and Child Oppositional Symptoms

**DOI:** 10.1007/s10578-023-01575-0

**Published:** 2023-07-21

**Authors:** Marie-Theres Klemp, Christina Dose, Judith Mühlenmeister, Julia Plück, Laura Wähnke, Manfred Döpfner

**Affiliations:** 1 Faculty of Medicine, School for Child and Adolescent Cognitive Behavior Therapy (AKiP), University of Cologne, University Hospital Cologne, Pohligstr. 9, 50969 Cologne, Germany; 2 Faculty of Medicine, Department of Child and Adolescent Psychiatry, Psychosomatics and Psychotherapy, University of Cologne, University Hospital Cologne, Robert-Koch-Str. 10, 50931 Cologne, Germany

**Keywords:** Attention-deficit hyperactivity disorder, Oppositional defiant disorder, Parenting, Longitudinal analysis, Randomized controlled trial

## Abstract

Research has pointed to both cross-sectional and longitudinal associations between parental internalizing symptoms and child externalizing symptoms. This study analyzed whether the association is mediated by negative parenting behavior in view of previous reports that both parental internalizing symptoms and child externalizing symptoms are related to parenting behaviors. Longitudinal data for the current analyses were derived from a randomized controlled trial on the efficacy of a web-assisted self-help intervention for parents of children with elevated levels of externalizing symptoms. Two different mediation models were analyzed, one using attention-deficit/hyperactivity disorder (ADHD) symptoms as the dependent variable and the other using oppositional defiant disorder (ODD) symptoms. Both models included parental internalizing symptoms as the independent variable, negative parenting behavior as a mediator, and study condition as a confounder. The longitudinal analyses support the mediating role of negative parenting behavior in the association between early parental internalizing symptoms and later child ODD symptoms.

**Supplementary Information:**

The online version contains supplementary material available at 10.1007/s10578-023-01575-0.

## Introduction

Externalizing disorders are common in childhood and represent one of the main reasons for referral to child and adolescent mental health services [1]. The most frequent externalizing disorders between the ages of 6 and 12 years are oppositional defiant disorder (ODD), with a prevalence of 4.9%, and attention-deficit hyperactivity disorder (ADHD), with a prevalence of 4.3% [2]. To improve the treatment of these disorders, deeper knowledge about their etiology and pathogenesis is needed.

The etiology of both ADHD and ODD is considered to be multifactorial [3, 4]. That is, both genetic and environmental factors seem to affect the development of these disorders. Regarding ADHD, genetic factors seem to play the most important role [5], while for ODD, environmental factors seem to be more determinant [6]. Previous research has shown that parental factors such as parental psychopathology and parenting behaviors can have a significant impact on the development and manifestation of ODD and ADHD symptoms in children [7–9].

Previous research has demonstrated positive associations of internalizing symptoms of the parents, such as anxiety and depressive symptoms, with child externalizing symptoms. While some studies reported positive longitudinal associations between parental internalizing symptoms at an early assessment point and child externalizing symptoms (e.g. ADHD or overarching externalizing symptoms) at a later assessment point [10–12], others found associations between early child externalizing symptoms (i.e. child inattention and oppositional-conduct problems) and later parental mood and stress, pointing at the reverse direction of effect [13, 14]. Moreover, some studies have indicated bidirectional associations for children with ODD, disruptive behavior and externalizing behavior in general [15–17].

To make these etiological considerations and related empirical findings useful for improving the prevention and treatment of externalizing behavior disorders, knowledge regarding the mechanisms through which parental internalizing symptoms exert their effects on child externalizing symptoms is crucial.

In this regard, the integrative model by Goodman and Gotlib [18] suggests four potential mechanisms: (1) genetic factors, (2) innate dysfunctional neuroregulatory mechanisms, (3) exposure to dysfunctional parental cognitions, behaviors, and affect, and (4) the stressful life context of the children. Especially the third factor might be of interest when deriving conclusions for treatment, as parental cognitions and behaviors are potentially modifiable. A more detailed presentation of the way in which parenting behaviors might affect child externalizing behaviors is provided by Patterson’s model of the family coercive process [19]. This model provides a theoretical framework to understand how disruptive behavior problems in children are shaped within the family environment [19]. According to this model, ongoing cycles of negative interactions between parents and children can lead to the mutual reinforcement and escalation of problematic behaviors. This cycle is characterized by coercive exchanges, where both parents and children engage in aversive and confrontational behaviors [19].

In line with these models, parental health and psychological well-being have been found to be related to parenting behavior [20]. Maternal symptoms of anxiety appear to be cross-sectionally related to less maternal warmth and less positive engagement [21]. Moreover, based on longitudinal data, mothers with depressive symptoms have been found to show a decline in positive parenting practices and to report less consistent parenting behavior, fewer positive parenting practices, and less warm and nurturing parenting behaviors [22, 23].

Previous research also points to a positive cross-sectional association of negative and inconsistent parenting behavior with children’s antisocial behavior [24]. Typically, the term negative parenting behavior refers to inconsistency, impulsivity and rigidity in parents’ behavior. Parents of children with externalizing symptoms (i.e. ODD, ADHD) have been shown to use more negative parenting practices, to be critical of their children, and to show less monitoring behavior in community samples [25, 24, 26–28]. Positive parenting behaviors, by contrast, comprise behaviors that serve to promote favorable parent-child interactions, and previous longitudinal studies have demonstrated protective effects of positive parent-child interactions in school, community, and clinical samples [29].

Studies examining positive and negative parenting behaviors as mediators of the effects of behavioral treatments hint at a greater importance of negative parenting behaviors for children’s behavior problems (i.e. ODD and ADHD) [30–34], suggesting that this association is particularly crucial [35, 32, 36, 37]. However, some longitudinal studies found that only positive parenting behaviors acted as mediators, or that a combination of positive and negative parenting behaviors exerted an impact [38–40].

Studies focusing directly on the mediation of the association between parental internalizing symptoms and child externalizing symptoms through parenting behaviors are rare. Dette-Hagenmeyer & Reichle [41] found in a nonclinical sample that mothers’ inconsistent use of discipline mediated the longitudinal association between maternal depressive symptoms and child ODD and hyperactive symptoms. Moreover, mediation analyses in a clinical sample suggest that parental affirmation [42] and negative parental emotional expressiveness [43] mediate the association between parental internalizing symptoms and child or adolescent externalizing symptoms.

Tom sum up, to the best of our knowledge, only few studies have directly explored how parenting practices could serve as mediators of the association between parental symptoms of depression, anxiety, and stress on the one hand, as well as the manifestation of externalizing symptoms in children on the other hand. Currently, no study has focused on the mediation of the association between parental internalizing symptoms and externalizing symptoms in children through parenting behaviors in a clinical sample of children with elevated levels of externalizing symptoms from a longitudinal perspective. In a first analysis based on cross-sectional data, we found that negative parenting behaviors mediated the association between parental internalizing symptoms and ODD symptoms, but not the association between parental internalizing symptoms and ADHD symptoms [44]. A possible explanation for this discrepancy might be that ADHD symptoms are determined more by genetic influences compared to ODD symptoms, for which environmental influences seem to play a greater role [45–47]. Since the development of both disorders is multifactorial, it is necessary to determine how crucial parental symptoms and parenting behaviors are in this process, how they interact, and to what extent they influence the child’s symptoms. Moreover, in the previous cross-sectional analysis, we detected a small indirect effect of parental internalizing symptoms on child ADHD symptoms through positive parenting behaviors. However, the direction of this effect was contrary to our expectation. While in line with our assumption, increased parental internalizing symptoms were associated with a lower level of positive parenting behaviors, in contrast to our hypothesis, a lower level of positive parenting behaviors was related to lower ADHD symptom severity [44].

The aim of the current study was to examine whether negative parenting behavior also mediates the longitudinal association between parental internalizing symptoms (that is, symptoms of depression, anxiety, and stress) and child externalizing symptoms (ADHD symptoms, ODD symptoms). Using data from three consecutive assessment points, we analyzed whether higher levels of parental internalizing symptoms (such as depression, anxiety, and stress) would predict higher levels of negative parenting behavior, which in turn would lead to more severe externalizing symptoms in the child. As our first set of cross-sectional analyses yielded different mediation effects in the models considering ADHD and ODD symptoms, respectively, as outcomes, we decided to also consider these two externalizing domains separately in the current longitudinal analyses. As in the analyses based on cross-sectional data, we hypothesized a significant mediation of the association between parental internalizing symptoms and child ODD symptoms through negative parenting behavior, but no such mediating effect when considering child ADHD symptoms as the outcome. Our previous cross-sectional analyses did not detect any reasonably interpretable mediating effects through positive parenting behaviors. Moreover, there seems to be a larger empirical basis for the mediation through negative parenting behavior compared to positive parenting behavior [48, 36, 35, 49, 25]. Accordingly, we decided to concentrate on mediating processes through negative parenting behavior in the current analyses.

To allow for a more fine-grained analysis of the mediating effect of negative parenting behavior on the association between different domains of internalizing symptoms and child externalizing symptoms, we subsequently considered separate models including parental symptoms of depression, anxiety, or stress, respectively. The findings of this research are intended to help to improve the treatment of externalizing behavior problems by providing information on putative etiological mechanisms.

## Methods

### Study Design and Participants

The data for the present analyses originate from a randomized controlled trial (RCT) on the efficacy of a twelve-month web-assisted self-help (WASH) program for parents of children with externalizing symptoms, that is, symptoms of ADHD and/or ODD [50]. The randomized controlled trial (RCT) compared three different conditions: the first one was treatment as usual (TAU), the second one was WASH in addition to TAU, and the third one was WASH along with telephone-based support in addition to TAU. Data for the present analyses were collected at baseline (T1), after three months (interim assessment; T2) and after six months (post-assessment, T3) in all three study conditions. Registration was carried out at the German Clinical Trials Register (identifier: DRKS00013456).

For participant recruitment, we sent study information to 5,015 pediatricians and child and adolescent psychiatrists throughout Germany, who were encouraged to register eligible families. The recruitment period lasted from December 2017 to February 2020. Families were eligible to participate in the RCT if they met the following criteria: (a) the child was aged between 6 and 12 years, (b) the referring health care provider had diagnosed the child with an externalizing behavior disorder or suspected such a diagnosis, and (c) the child showed an elevated level of externalizing symptoms, as indicated by a clinician based on the semi-structured German-language Clinical Parent Interview for Externalizing Disorders in Children and Adolescents (ILF-EXTERNAL) [50–52], which was conducted by telephone. In particular, symptoms were considered as significantly elevated if the mean score on any of the externalizing scales or the total score on the ILF-EXTERNAL was more than 1.5 standard deviations above the mean score of a representative normative sample on the corresponding scale of a parent-rated questionnaire containing the same items [53]. Clinicians were required to assess the parent interview and determine if the child demonstrated five or more symptoms of inattention, four or more symptoms of hyperactivity-impulsivity, eight or more symptoms of ADHD (either inattentive or hyperactive-impulsive), or four or more symptoms of ODD. The diagnostic criteria were evaluated by clinicians and resulted in the following diagnoses: 39.22% of the children did not meet diagnostic criteria for any diagnosis, 18.10% of the children were diagnosed with the predominantly inattentive subtype of ADHD, 6.90% with the predominantly impulsive/hyperactive subtype of ADHD, 9.05% with the combined type of ADHD, 8.62% of the children fulfilled diagnostic criteria for ODD, and 18.10% for any ADHD diagnosis combined with ODD.

Children who met diagnostic criteria for a serious mental illness or autism spectrum disorder, or who needed to be hospitalized as determined by the referring healthcare provider, were excluded from the study. The article uses the terms “parents” or "mother/father" to refer to all individuals who serve as the primary caregivers for the child and are expected to take on parenting responsibilities.

### Measures

All measures were completed by the participating parents and processed online. There was no study-related face-to-face contact with any of the participants or parents.

#### Depression Anxiety Stress Scales (DASS), Parent Rating

The German version of the DASS [37–40] comprises 42 items assessing the extent to which symptoms of depression, anxiety, and stress have been experienced in the preceding week. The questionnaire uses a four-point scale (ranging from 0 to 3) to rate symptoms related to depression, anxiety, and stress. The item scores can be combined to a total score and three 14-item subscales. Both exploratory and confirmatory factor analyses have yielded support for the factor structure of the DASS [54]. Moreover, the DASS subscales have shown high internal consistency (α ≥ .81) and convergent validity [54]. In the present sample, all subscales and the total score showed good to very good internal consistency at all three measurement time points (Cronbach’s α for the total score: .95 to .97, Cronbach’s α for all three subscales: .85 to .93).

#### Questionnaire for Positive and Negative Parenting Behavior (PNPQ), Parent Rating

The PNPQ (German: “Fragebogen zum positiven und negativen Erziehungsverhalten” [55]) assesses positive and negative parenting behaviors. It is based on the Management of Children’s Behavior Scale (MCBS [56, 57]) and the Parent Practices Scale (PPS [58]). Furthermore, it encompasses newly created items that address specific aspects of behavioral parent training, such as the management of family rules. For the current analyses, we only applied the 17-item subscale on negative parenting behaviors. The items are answered using a four-point scale ranging from 1 (never) to 4 (very often/most of the time). To obtain scale scores, the average of the associated item scores was taken. The MCBS, PPS, and FPNE have demonstrated good psychometric properties in terms of internal consistency and construct validity [55, 56, 58]. Moreover, the MCBS has been shown to be sensitive to change [56]. In the present sample, Cronbach’s alpha was .63 to .67 for the negative parenting scale across all three measurement time points.

#### German Symptom Checklists for Attention-Deficit Hyperactivity Disorder (SCL-ADHD) and Disruptive Behavior Disorders (SCL-DBD), Parent Rating

The SCL-ADHD (German: “Fremdbeurteilungsbogen für Aufmerksamkeitsdefizit-/Hyperaktivitätsstörungen [53]) and the SCL-DBD (German: “Fremdbeurteilungsbogen für Störungen des Sozialverhaltens [53]) capture items of ADHD and disruptive behavior disorders, respectively, according to the DSM-5 and ICD-10. From the SCL-DBD, we only used the ODD subscale, which is suitable for the age group under study. The 18 symptom-related items of the SCL-ADHD and the eight items of the SCL-DBD ODD subscale are rated on a four-point Likert-type scale ranging from 0 (not at all) to 3 (very much/particularly severe). To obtain the ADHD score and the ODD score, the item scores were averaged. The SCL-ADHD and SCL-DBD have been shown to be valid in terms of their factor structure and reliable in terms of internal consistency [53, 59–61]. In the present sample, Cronbach’s alpha was .89 to .91 for the SCL-ADHD total score and .88 to .90 for the SCL-DBD score across the three measurement time points.

### Statistical Analyses

To examine whether negative parenting behavior mediates the longitudinal association between parental internalizing symptoms and ADHD or ODD symptoms, respectively, we conducted mediation analyses using the SPSS macro PROCESS [62], which estimates the model parameters based on ordinary least squares (OLS) regression.

A simple mediation model assumes that an independent variable (*X*) influences a dependent variable (*Y*) through a mediator variable (*M*) [63]. In this model, the direct effect of X on Y when controlling for M (c’) and the indirect effect of X on Y through the mediator variable M (ab) add up to the total effect (c) [63]. The indirect effect (*ab*) can be expressed as the product of two paths, namely the effect of *X* on *M* (*a*) and the effect of *M* on *Y* after controlling for the effect of *X* (*b*), and can be tested for significance [63]. In the context of statistical analysis, a mediator is a variable that helps explain the underlying mechanism or process through which the independent variable affects the dependent variable.

**Fig. 1:**
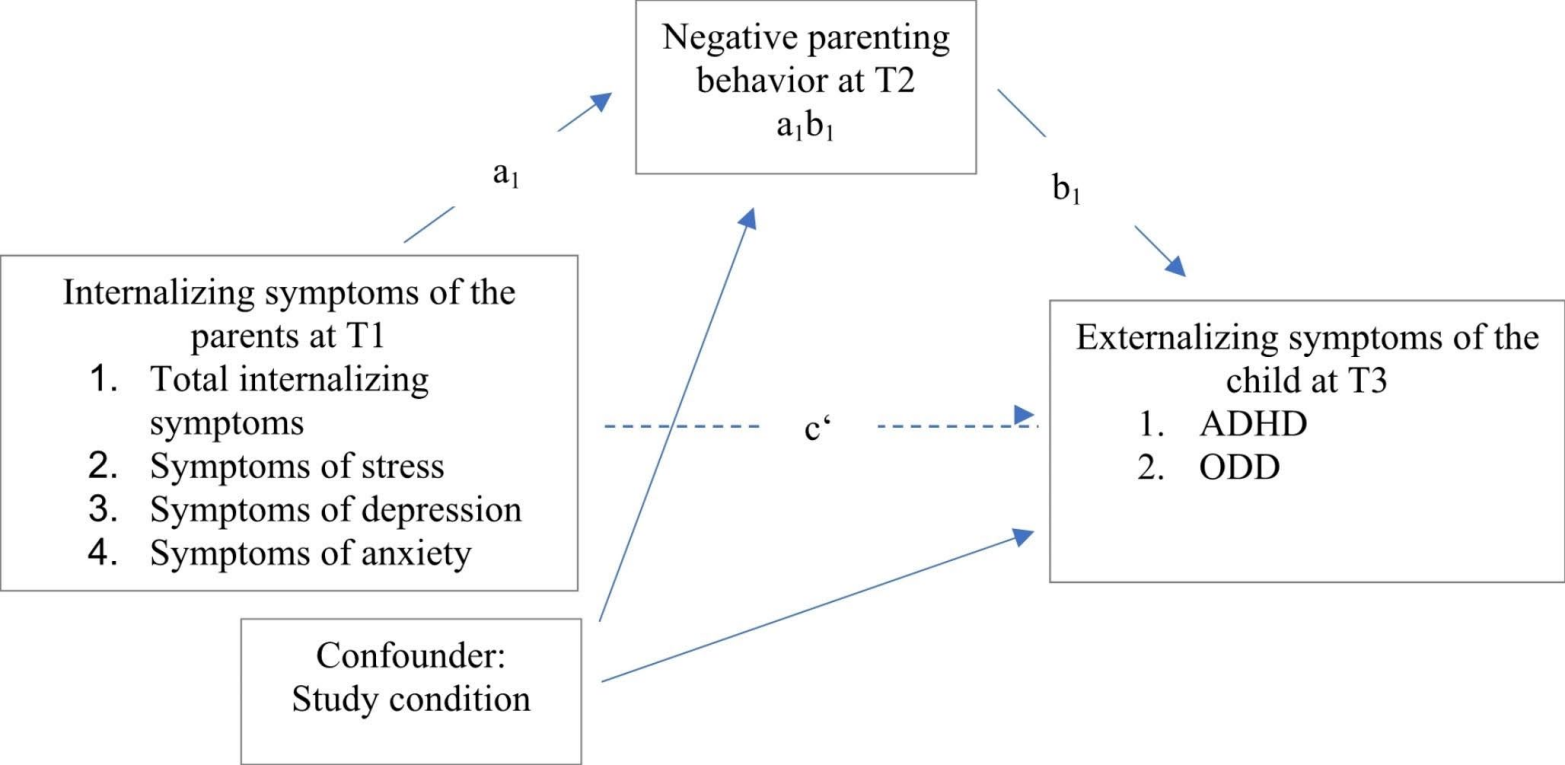
Single Mediator Model for the Mediation of the Association of Parental Internalizing Symptoms and Child Externalizing Symptoms Through Negative Parenting Behavior. ***Note.*** In total, eight different models were considered: Either child ADHD symptoms or child ODD symptoms at T3 (post-assessment) were considered as the dependent variable, and either total parental internalizing symptoms (symptoms of depression, anxiety, and stress combined), symptoms of depression, symptoms of anxiety, or symptoms of stress at T1 (baseline) as the independent variable. All models used negative parenting behavior at T2 (three-month interim assessment) as the mediator. The results for the models including symptoms of depression, symptoms of anxiety and symptoms of stress as the independent variable are provided in the online supplement. Study condition was included as a confounding variable in all models. ADHD = attention-deficit/hyperactivity disorder, ODD = oppositional defiant disorder

In the present study, we set up eight separate single mediator models, modelling either child ADHD symptom severity or child ODD symptom severity as the dependent variable and either parental symptoms of anxiety, symptoms of depression, symptoms of stress, or total parental internalizing symptoms as the independent variable. In each model, negative parenting behavior was used as a mediator (see Fig. 1). Moreover, to control for the likely confounding influence of the study condition, we included this variable as a covariate in our models. That is, the study condition was included as a predictor of both the mediator (negative parenting behaviors) and the dependent variable (ADHD symptom severity or ODD symptom severity, respectively).

To account for the causal relationships that are generally hypothesized by mediation models [62–64], we assessed parental internalizing symptoms (independent variable), negative parenting behavior (mediator variable) and child externalizing symptoms (dependent variable) in a consecutive order. That is, we examined the hypothetical model that a higher level of parental internalizing symptoms at baseline (T1) would be associated with a higher level of negative parenting behavior at the interim assessment (T2), which would in turn lead to a higher severity of child externalizing symptoms at post-assessment (T3).

To decide on the significance of the mediation effect, as recommended by Hayes [62], we regarded the significance of the product *ab* but not the significance of the single paths *a* and *b*. We report unstandardized regression coefficients as well as percentile bootstrap confidence intervals (10,000 iterations [62]), with estimates being considered as statistically significant if their 95% confidence interval does not include zero. We computed completely standardized total, direct, and indirect effects. Completely standardized effects are obtained by dividing the unstandardized effects by the standard deviation of the dependent variable and multiplying them by the standard deviation of the independent variable [62]. We also regarded the percentage of variance in the mediator explained by the independent variable and the covariate taken together and the percentage of variance in the dependent variable explained by the independent variable, the covariate and the mediator taken together to evaluate how well the hypothetical models fit the data [62].

To obtain an impression of the influence of potentially confounding variables (apart from study condition, which was included as covariate into the main analyses), we performed an additional set of analyses. These analyses included baseline values of the respective dependent variable (ADHD or ODD) and of the mediator (negative parenting behavior) (cf. [65]), child’s sex and child age as additional covariates to control for their influence. Moreover, the ADHD model included post-assessment ODD symptoms as additional covariate, while the ODD model additionally controlled for post-assessment ADHD symptoms to account for the mutual influences of these variables.

## Results

### Sample Characteristics

**Fig. 2:**
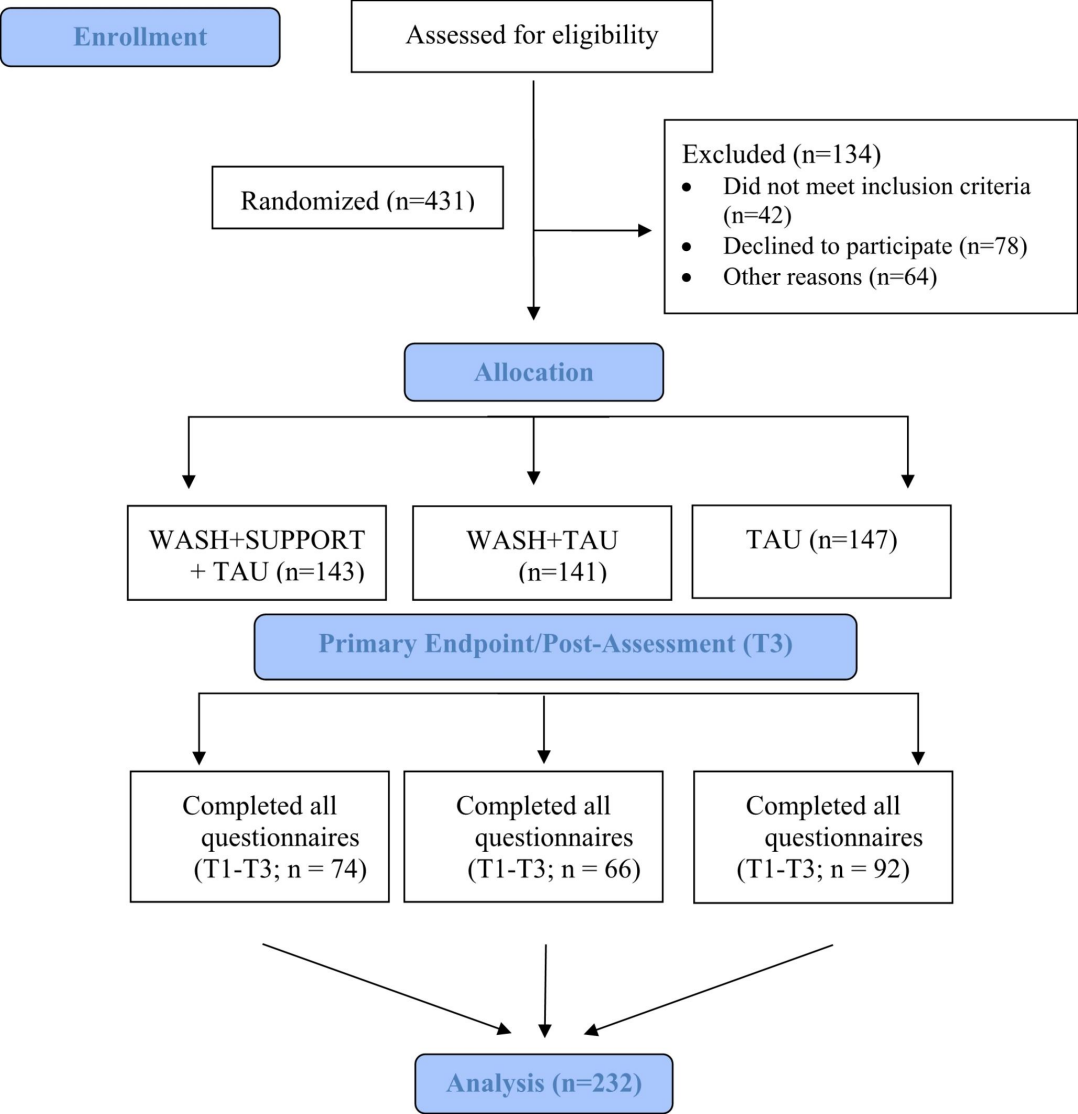
Selection of Sample of Analysis. ***Note.*** WASH = web-assisted self-help, SUPPORT = telephone-based support, TAU = treatment as usual, T1 = baseline assessment, T2 = three-month interim assessment, T3 = post-assessment

Between January 2018 and March 2020, N = 565 participants were registered by pediatricians and child and adolescent psychiatrists. Of these, 431 families were eligible for the study, agreed to participate, and were randomized to one of the three study conditions. The present analyses included the data of n = 232 families who completed the online questionnaires at all three measurement time points (see Fig. 2).

The mean age of these children was 9.42 years (*SD* = 1.71) and 82.33% were male. In addition to the diagnoses mentioned above from the interviews, the diagnoses of the children as indicated by the referring physicians were as follows: 55.17% ADHD (F90.0), 14.66% hyperkinetic conduct disorder (F90.1), 4.31% other specified behavioral and emotional disorders (F98.8), 0.86% ADHD, other type or unspecified type (F90.8; F90.9), and 0.43% ODD (F91.3). In addition, the physicians who referred the children assigned a tentative diagnosis of ADHD in 24.57% of the cases. At the outset of the study, approximately half of the children (51.72%) were taking medication for ADHD and 22.41% of the parents reported that their child was currently receiving psychotherapy. In the majority of cases (88.79%), the biological mother of the child with externalizing behavior problems participated in the study; 7.76% of the participants were biological fathers, 3.02% were adoptive mothers and 0.43% were other caregivers. On average, the participating parents were 42.11 years old (*SD* = 5.63). Most of the children (73.28%) lived with both of their biological parents, 10.78% lived with their mother only, 0.86% lived with their father only, 11.21% lived with their mother and her partner, 0.43% lived with their grandparents or other relatives, and 3.45% of families reported other constellations (e.g., weekly rotation principle, foster care). Descriptive statistics for child externalizing behavior problems, negative parenting behavior and parental internalizing symptoms at T1, T2 and T3 can be found in the online supplement (see Table S1).

### Mediation Analysis

The mediation analyses yielded both a significant total effect and a significant direct effect after controlling for negative parenting behavior (mediator) in the model including parental internalizing symptoms as the independent variable and ADHD symptoms as the dependent variable. In this model, parental internalizing symptoms were significantly and positively associated with negative parenting.

The association between negative parenting behavior and child ADHD symptoms was non-significant. Likewise, the indirect effect of parental internalizing symptoms on child ADHD symptoms through negative behavior was non-significant (see Table 1).

**Table 1:** Unstandardized Regression Coefficients, Bootstrap Confidence Intervals, and Model Information for the Mediation Model

	Outcome							
	ADHD				ODD			
	Coeff.	Bootstrap SE	95% Bootstrap CI	Completely Standardized effect	Coeff.	Bootstrap SE	95% Bootstrap CI	Completely Standardized effect
*a*_1_	0.22*	0.04	0.14; 0.30		0.22*	0.04	0.14; 0.30	
*b*_1_	0.13	0.13	-0.11; 0.38		0.55*	0.17	0.22; 0.90	
*a*_1_ *b*_1_	0.03	0.03	-0.02; 0.09	0.02	0.12*	0.05	0.04; 0.22	0.07
*c*‘	0.33*	0.09	0.15; 0.49	0.25	0.19	0.12	-0.05; 0.44	0.11
*c*	0.36*	0.08	0.19; 0.52	0.27	0.31*	0.11	0.09; 0.53	0.18

***Note***. sample size *n* = 232. *a*_1_: parental internalizing symptoms (i.e., symptoms of depression, anxiety, and stress) at baseline ◊ negative parenting behavior at three-month interim assessment, *b*_1_: negative parenting behavior at three-month interim assessment ◊ outcome at post-assessment, *a*_1_*b*_1_: indirect effect of parental internalizing symptoms on outcome through negative parenting behavior, *c‘*: direct effect of parental internalizing symptoms on outcome, *c*: total effect of parental internalizing symptoms on outcome, ADHD = attention-deficit/hyperactivity disorder, ODD = oppositional defiant disorder, Coeff. = unstandardized regression coefficient, SE = standard error, CI = confidence interval. * significant coefficient (95% CI). The standard errors and confidence intervals for the total effects were determined without the use of bootstrap samples. Study condition was included as a confounding variable in both models

This present model, 13% of the variance in negative parenting behavior was accounted for by parental internalizing symptoms and study condition (covariate). Additionally, parental internalizing symptoms, study condition (covariate) and the mediator taken together explained about 8% of the variance in child ADHD symptoms.

The model comprising the total score for parental internalizing symptoms as the independent variable and child ODD symptoms as the dependent variable yielded a significant total effect but no significant direct effect. In this model, we detected both a significant positive association between parental internalizing symptoms and negative parenting behavior and a significant positive association between negative parenting behavior and child ODD symptoms. Furthermore, there was a significant indirect effect of parental internalizing symptoms on the severity of child ODD symptoms through negative parenting behavior. This means that higher levels of parental internalizing symptoms were associated with increased negative parenting behavior, which in turn was associated with greater severity of ODD symptoms in children. The corresponding completely standardized indirect effect was 0.07. In other words, when parents differ by one unit in their internalizing symptoms, their children’s ODD symptom severity differs by approximately one tenth of a standard deviation due to the indirect effect through negative parenting behavior. In this model, parental internalizing symptoms, study condition and negative parenting behavior taken together accounted for about 8% of the variance in ODD symptoms.

When the DASS subscales (i.e., parental symptoms of depression, anxiety, and stress) were considered separately as independent variables, overall, the results were comparable to those for the models including a composite score for parental internalizing symptoms (see Table S2 in the online supplement).

Regarding the models which controlled for additional potential confounders (baseline values of the dependent variable, baseline values of the mediator, child age, child sex, ODD symptoms in the ADHD model/ADHD symptoms in the ODD model), the total effect was non-significant for both models, which was contrary to the results for our original models. According to Hayes (2018), a significant total effect is not a prerequisite for detecting mediation. However, the analyses for these more complex models did not yield any significant indirect effects. That is, contrary to the results for our original analyses, the indirect effect of parental internalizing symptoms on child ODD symptoms through negative parenting behavior just failed to reach significance (a1b1 = 0.007, 95%-Bootstrap-CI = [-0.009, 0.04], completely standardized indirect effect = 0.004; see Table S3 in the online supplement).

## Discussion

The aim of the current study was to examine the mediating role of negative parenting behavior in the frequently reported longitudinal association between parental internalizing symptoms (i.e., depression, anxiety, and stress) and child externalizing symptoms (ADHD symptoms and ODD symptoms) in a sample of school-age children with elevated levels of externalizing behavior problems. Although several studies have revealed both cross-sectional and longitudinal associations between parental internalizing symptoms and child ADHD and ODD symptoms [7–12], suggesting that parental psychopathology might play a role in the etiology of both disorders, little empirical research has focused on the mechanisms through which parental internalizing symptoms might affect child externalizing symptoms. Based on the integrative model on the transmission of risk for psychopathology by Goodman & Gotlib [18], we examined negative parenting behaviors as one putative mechanism. Previous research has yielded support for the single paths that constitute our model, that is, besides for the association between parent and child symptoms, for the associations between parental internalizing symptoms and negative parenting behaviors [20, 21, 23, 8] and for the associations between negative parenting behaviors and child externalizing symptoms [24, 26]. However, to our knowledge, no study has analyzed a comprehensive mediation model combining these paths in a sample of children with elevated levels of externalizing behavior problems. The analyses yielded significant associations between parental internalizing symptoms at the start of the study and the severity of both child ADHD symptoms and child ODD symptoms at post-assessment. Furthermore, our results demonstrated that negative parenting behaviors (assessed at an interim assessment point) mediated the association between parental internalizing symptoms and child ODD symptoms, supporting the hypothesis that parental behavior is one of the mechanisms through which parental symptoms might affect child symptoms, as suggested by Goodman & Gotlib [18]. However, no such indirect effect could be detected in the model including ADHD symptoms as the dependent variable. In other words, in line with our hypotheses, a higher level of early parental internalizing symptoms was followed by a higher level of negative parenting behavior, which in turn predicted a higher level of later child ODD symptoms. When we included a number of additional putative confounders into this model, the indirect effect through negative parenting behavior just failed to reach significance. However, this might also be due to reduced power to detect a significant indirect effect in this more complex model. Hence, we conclude that our study provides limited evidence for indirect effect of parental internalizing symptoms on child externalizing symptoms through negative parenting behavior. Whether this effect persists in more complex models, including more confounders, could be subject to further studies using larger samples. When setting up separate models for parental depression, anxiety, and stress symptoms as independent variables instead of considering a composite score for parental internalizing symptoms, the results remained very similar. That is, the analyses of all models yielded significant associations of depression, anxiety, or stress symptoms, respectively, with both child ADHD and ODD symptoms. Again, no significant indirect effect through negative parenting behavior was detected in the models employing child ADHD symptoms as the dependent variable. However, the respective indirect effect was significant in all models using child ODD symptoms as the dependent variable.

To sum up, in line with our expectations, the present findings underline the role of negative parenting behavior in mediating the association of parental internalizing symptoms with child ODD symptoms, but not child ADHD symptoms. Thus, the findings for the ODD model is in line with the model of transmission of risk for psychopathology by Goodman & Gotlib [18], which assumes that a mediation through parental behaviors is one of several mechanisms through which parental symptoms might exert their effects on child symptoms, and also with previous empirical findings. In particular, this finding corresponds to the results of our previous mediation analysis based on cross-sectional data [44]. Moreover, it is in line with the results of previous mediation studies which demonstrated that the longitudinal association between parental depressive symptoms and child externalizing symptoms was mediated by parental nurturance and by parental rejection in a community sample [66], that the cross-sectional association between maternal internalizing symptoms and child externalizing symptoms were mediated by maternal affirmation in a clinically referred sample [43], and that the longitudinal relation between parental depressive symptoms and child maladjustment was mediated by the use of inconsistent discipline in a nonclinical sample [41]. Interestingly, the latter two studies found different patterns in mothers and fathers. While Bellini et al. were only able to establish the above-mentioned mediating effect for mothers, but not for fathers [43], Dette-Hagenmeyer et al. report that positive parenting was found to be an additional mediator of the effects of paternal, but not maternal, depressive symptoms on child adjustment [41]. However, due to the small number of fathers in our sample, we were not able to examine whether the results for our model differ between mothers and fathers.

Of note, in all models, parental internalizing symptoms, study condition (covariate) and negative parenting behavior taken together only accounted for a small amount of the variance in child ADHD or ODD symptoms, respectively, pointing to a rather poor data fit of the hypothesized models. Thus, there are likely other (additional) variables that mediate the association between parental internalizing and child externalizing symptoms, such as a common genetic predisposition and/or environmental factors, stressful life events or personality traits like neuroticism [67, 48, 68–71].

The finding that a significant indirect effect only emerged in the ODD model and not in the ADHD model might be attributable to the fact that ADHD symptoms are more strongly determined by biological or genetic factors [72, 73, 70, 5, 74]. The development and maintenance of oppositional and defiant symptoms, by contrast, is often explained by environmental factors. For instance, the coercive family process model by Patterson assumes a mutual reinforcement of dysfunctional parenting practices and child troublesome behaviors [19]. This theory is supported by empirical findings [68], and in our analyses it might particularly explain the association between negative parenting behaviors and child ODD symptoms.

Taken together, the results of our mediation analyses and previous empirical findings on the mediation of the effects of parental internalizing symptoms on child externalizing symptoms or child maladjustment point at the important role of parenting behaviors as mediator. In our study, this mediation process related to child ODD symptoms as an outcome, but not to child ADHD symptoms. For clinical practice, these findings would suggest that if parents demonstrate internalizing symptoms, they should receive particular counselling regarding their parenting behaviors, as these are one of several putative mechanisms mediating between their symptoms and the child’s externalizing symptoms (or especially ODD symptoms, as the present study suggests). Such an approach is usually taken in behavioral parent training interventions.

The present study has several strengths and limitations. A particular strength is that we were able to consider longitudinal data, with the components of the model being assessed in a consecutive order. This is often recommended to account for the causality that is generally hypothesized in mediation models. Moreover, in contrast to most previous studies, we considered a clinical sample with a good sample size and a homogeneous age group.

On the other hand, some limitations have to be considered when interpreting the results. First, all measures were completed by the participating parents; thus, the respective ratings might potentially be biased by socially desirable responding and by simulation or dissimulation tendencies. Previous research on the association between self-reported and observer-reported parenting behavior only demonstrated a significant correlation for parental warmth, and found no significant associations between direct observations of parental control practices, including inconsistency, and self-judgment of these behaviors [75]. Another study reported no significant association between parent and child perceptions of parenting behavior [76]. Moreover, pertaining directly to mediation analyses, a previous study was unable to establish observed parent-child interactions as a mediator of the association between parental depressive symptoms and child internalizing and externalizing symptoms [77]. The previous study, however, had various limitations, such as potentially inadequate statistical power to identify a mediating effect [77]. Moreover, as all measures were rated by the parents, common method bias is conceivable. We performed Harman’s single-factor test for common method bias, which did not point at the presence of such bias in the current data. However, as the sensitivity of this test has been criticized [78], and as we did not control for common method bias in our analyses, we cannot completely rule out the possibility that such bias might have had an influence. Given the aforementioned limitations associated with the ratings of all measures by the same rater, and to account for the different previous findings for data from different informants, future studies should incorporate different sources of information (e.g., clinical ratings and observations of parental behavior).

Second, we did not analyze potential moderators of the mediation effects (e.g. parental ADHD). A previous study revealed that mothers with ADHD symptoms demonstrated more psychiatric and occupational problems, more dysfunctional cognitions (lower self-esteem, more external locus of control) and less effective parenting practices than mothers without ADHD symptoms [79]. Unfortunately, we did not assess parental externalizing psychopathology in the present study.

Third, the vast majority of the participating parents were mothers, which might limit the ability to generalize the results to the mediation of the associations between fathers’ potential psychopathology and child symptoms through paternal parenting behavior. As outlined above, given the limited number of fathers in our sample (n = 18), we were not able to compare results between mothers and fathers. Similarly, the sample only comprised a small proportion of girls (17.67%), again limiting the generalizability of the findings.

Fourth, another study limitation is the broad age range (6–12 years) considered in this study. It might be possible that the associations examined in this study differ between age groups. For instance, it is conceivable that the association between parenting behaviors and symptom severity is stronger in younger children. This question could be subject to future research.

Fifth, data were collected during the COVID-19 pandemic. Pandemic-related stress may have influenced both the parents’ and the children’s symptoms and biased the results. In line with this, a recent study shows that the pandemic led to interruptions in psychological treatment and had an impact on families due to COVID-19-related stress [80].

Sixth, also we conducted an additional set of analyses to obtain an impression of the influence of potentially confounding variables, we cannot rule out that there might have been other or additional confounders. For example, it is conceivable that family socioeconomic status has an influence on the results. Unfortunately, our collected data does not include a suitable variable that describes the family socioeconomic status. As outlined above, contrary to our original analyses, in the more complex model controlling for more confounders the indirect effect of parental internalizing symptoms on child externalizing symptoms through negative parenting behavior just failed to reach significance. On the one hand, this might indicate the absence of such an indirect effect. However, given that the indirect effect in this model was close to significance and that this model included a number of more variables than the original ODD model, the non-significance of this effect might also be due to reduced power. Thus, we conclude that our analyses provide limited evidence for the indirect effect of parental internalizing symptoms on child externalizing symptoms through negative parenting behavior.

Preferably, future longitudinal studies should concentrate on children at risk of developing externalizing behavior symptoms to be able to more closely approach the causality hypothesized in mediation models. Reciprocal relationships are, of course, also conceivable and could be considered in further research projects.

### Summary

Previous research has revealed associations between parental internalizing symptoms and child externalizing symptoms, as well as between parenting behaviors and child externalizing symptoms. Only a few studies have investigated, how parenting practices might act as mediators in the relationship between internalizing parental symptoms and externalizing symptoms in children. Moreover, an earlier analysis by our research group found that negative parenting behaviors mediated the association between parental internalizing symptoms (symptoms of depression, anxiety, and stress) and oppositional-defiant symptoms of the child. However, this finding was limited by the use of cross-sectional data. Thus, the present study examined whether negative parenting behavior also mediates the longitudinal association between early parental internalizing symptoms and later child externalizing symptoms (i.e., symptoms of attention-deficit/hyperactivity disorder [ADHD] and oppositional defiant disorder [ODD]). Data from 232 parents of children (6–12 years) with elevated levels of ADHD and ODD symptoms were collected at three consecutive assessment points during a randomized controlled trial on the efficacy of a web-assisted, parent-directed self-help intervention. Two mediation models were examined: In both models, parental internalizing symptoms at baseline were modeled as the independent variable and negative parenting behavior was modeled as a mediator. One model considered child ADHD symptoms at post-assessment as the dependent variable and the other model considered child ODD symptoms at post-assessment. Moreover, both models controlled for the possibly confounding influence of study condition. Regression analyses yielded a significant indirect effect of early parental internalizing symptoms on later child ODD symptoms through negative parenting behavior (assessed at an interim assessment point). No mediation effect was found for the model including child ADHD symptoms as the dependent variable. The longitudinal results provide evidence for the involvement of negative parenting behavior in mediating the correlation between parental internalizing symptoms and the severity of child ODD symptoms. This result is limited by the fact that this indirect effect just fails to reach significance in a model which controls for a number of additional potential confounders, which might, however, also be due to power issues. The consecutive assessment of the variables in this model accounts for the causality that is usually assumed by mediation models. The different findings between the two models might be explained by the fact that ODD symptoms are more strongly determined by environmental factors.

## Electronic Supplementary Material

Below is the link to the electronic supplementary material

Supplementary Material 1

## Data Availability

Data and materials are available on reasonable request.
