# Supplementary material for: Negative Parenting Mediates the Longitudinal Association between Parental Internalizing Symptoms and Child Oppositional Symptoms
Source: Child Psychiatry Hum Dev. 2023 Jul 21;56(2):1–13. doi: 10.1007/s10578-023-01575-0 (PMC11928399; doi:10.1007/s10578-023-01575-0)
Supplement: Supplementary file 1 — Supplementary Material 1 [file 10578_2023_1575_MOESM1_ESM.docx]

**Supporting Information for *Negative parenting mediates the longitudinal association between parental internalizing symptoms and child oppositional symptoms*** by Klemp et al. 2023

**Table S1**

*Descriptive Statistics for Child Externalizing Behavior Problems, Negative Parenting Behavior and Internal Parental Psychopathology*

| Measure | Measurement Time Point | Scale/Subscale | *M* | *SD* | Range (theoretical) |
| --- | --- | --- | --- | --- | --- |
| Symptom Checklists for ADHD and  DBD (SCL-ADHD, SCL-DBD) | T1 | ADHD | 1.75 | 0.51 | 0.40 – 2.90 (0-3) |
|  |  | ODD | 1.49 | 0.68 | 0.00 – 3.00 (0-3) |
|  | T2 | ADHD | 1.50 | 0.52 | 0.30 – 3.00 (0-3) |
|  |  | ODD | 1.34 | 0.66 | 0.00 – 2.88 (0-3) |
|  | T3 | ADHD | 1.42 | 0.53 | 0.25 – 2.85 (0-3) |
|  |  | ODD | 1.25 | 0.70 | 0.00 – 3.00 (0-3) |
| Depression Anxiety Stress Scales (DASS) | T1 | Stress | 0.78 | 0.47 | 0.00 – 2.50 (0-3) |
|  |  | Anxiety | 0.51 | 0.39 | 0.00 – 2.14 (0-3) |
|  |  | Depression | 0.55 | 0.40 | 0.00 – 2.36 (0-3) |
|  |  | Total | 0.61 | 0.40 | 0.02 – 2.26 (0-3) |
|  | T2 | Stress | 0.69 | 0.49 | 0.00 – 2.57 (0-3) |
|  |  | Anxiety | 0.45 | 0.44 | 0.00 – 2.14 (0-3) |
|  |  | Depression | 0.46 | 0.40 | 0.00 – 2.21 (0-3) |
|  |  | Total | 0.53 | 0.43 | 0.00 – 2.26 (0-3) |
|  | T3 | Stress | 0.65 | 0.53 | 0.00 – 2.93 (0-3) |
|  |  | Anxiety | 0.43 | 0.47 | 0.00 – 2.71 (0-3) |
|  |  | Depression | 0.43 | 0.43 | 0.00 – 2.64 (0-3) |
|  |  | Total | 0.50 | 0.46 | 0.00 – 2.76 (0-3) |
|  | T1 | Negative parenting behavior | 1.94 | 0.30 | 1.24 – 2.82 (1-4) |
| Questionnaire for Positive and Negative Parenting (FPNE) | T2 | Negative parenting behavior | 1.84 | 0.27 | 1.18 – 2.47 (1-4) |
|  | T3 | Negative parenting behavior | 1.83 | 0.29 | 1.00 – 2.82 (1-4) |

**Note.** Sample size n = 232; ADHD = attention-deficit/hyperactivity disorder, ODD = oppositional defiant disorder.

**Table S2**

*Single Mediator Models for the Mediation of the Impact of Parental Symptoms of Depression, Anxiety and Stress on Child Externalizing Symptoms Through Negative Parenting Behavior*

|  |  | Dependent variable | | | | | | | |
| --- | --- | --- | --- | --- | --- | --- | --- | --- | --- |
|  |  | ADHD | | | | ODD | | | |
| Independent variable |  | Coeff. | Bootstrap SE | 95%  Bootstrap CI | Completely  stand. effect | Coeff. | Bootstrap SE | 95%  Bootstrap CI | Completely  stand. effect |
| Parental symptoms  of depression | *a*_1_ | 0.19* | 0.04 | 0.12; 0.29 |  | 0.19* | 0.04 | 0.12; 0.29 |  |
|  | *b*_1_ | 0.14 | 0.13 | -0.10; 0.39 |  | 0.57* | 0.17 | 0.25; 0.91 |  |
|  | *a*_1_*b*_1_ | 0.03 | 0.03 | -0.02; 0.08 | 0.02 | 0.11* | 0.04 | 0.04; 0.21 | 0.06 |
|  | *c*‘ | 0.34* | 0.08 | 0.17; 0.50 | 0.26 | 0.17 | 0.13 | -0.08; 0.41 | 0.10 |
|  | *c* | 0.37* | 0.08 | 0.20; 0.53 | 0.28 | 0.28* | 0.11 | 0.06; 0.50 | 0.16 |
| Parental symptoms  of anxiety | *a*_1_ | 0.21* | 0.04 | 0.13; 0.29 |  | 0.21* | 0.04 | 0.13; 0.29 |  |
|  | *b*_1_ | 0.16 | 0.13 | -0.08; 0.42 |  | 0.57* | 0.17 | 0.24; 0.91 |  |
|  | *a*_1_*b*_1_ | 0.03 | 0.03 | -0.02; 0.09 | 0.02 | 0.12* | 0.04 | 0.05; 0.21 | 0.07 |
|  | *c*‘ | 0.30* | 0.09 | 0.11; 0.47 | 0.22 | 0.17 | 0.12 | -0.06; 0.42 | 0.10 |
|  | *c* | 0.33* | 0.09 | 0.16; 0.50 | 0.24 | 0.29* | 0.12 | 0.06; 0.52 | 0.16 |
| Parental symptoms  of stress | *a*_1_ | 0.19* | 0.04 | 0.12; 0.27 |  | 0.19* | 0.04 | 0.12; 0.27 |  |
|  | *b*_1_ | 0.14 | 0.13 | -0.11; 0.39 |  | 0.54* | 0.17 | 0.20; 0.89 |  |
|  | *a*_1_*b*_1_ | 0.03 | 0.03 | -0.02; 0.08 | 0.02 | 0.10* | 0.04 | 0.04; 0.19 | 0.07 |
|  | *c*‘ | 0.26* | 0.08 | 0.10; 0.41 | 0.23 | 0.18 | 0.11 | -0.03; 0.39 | 0.12 |
|  | *c* | 0.29* | 0.07 | 0.14; 0.43 | 0.25 | 0.28* | 0.10 | 0.09; 0.47 | 0.19 |

***Note.*** Sample size *n* = 232*.* *a*_1_: independent variable 🡪 negative parenting behavior, *b*_1_: negative parenting behavior 🡪 dependent variable, *a*_1_*b*_1_: indirect effect of independent variable on dependent variable through negative parenting behavior, *c*‘: direct effect of independent variable on dependent variable, *c*: total effect of independent variable on dependent variable, ADHD = attention-deficit/hyperactivity disorder, ODD = oppositional defiant disorder, Coeff. = unstandardized regression coefficient, SE = standard error, CI = confidence interval. * significant coefficient (95% CI). The standard errors and confidence intervals for the total effects were determined without the use of bootstrap samples. Study condition was included as a confounder variable in all models.

**Table S3**

|  | \| Outcome \| \| \| \| \| \| \| \| \| --- \| --- \| --- \| --- \| --- \| --- \| --- \| --- \| \| ADHD \| \| \| \| ODD \| \| \| \| \| Coeff. \| Bootstrap SE \| 95%  Bootstrap CI \| Completely  Standardized effect \| Coeff. \| Bootstrap SE \| 95%  Bootstrap CI \| Completely  Standardized effect \| | | | | | | | |
| --- | --- | --- | --- | --- | --- | --- | --- | --- | --- | --- | --- | --- | --- | --- | --- | --- | --- | --- | --- | --- | --- | --- | --- | --- | --- | --- | --- | --- | --- | --- | --- | --- |
| *a*_1_ | 0.06 | 0.04 | -0.02; 0.14 |  | 0.04 | 0.04 | -0.03; 0.12 |  |
| *b*_1_ | 0.23* | 0.10 | 0.04; 0.43 |  | 0.18 | 0.16 | -0.13; 0.50 |  |
| *a*_1_*b*_1_ | 0.01 | 0.01 | -0.004; 0.04 | 0.01 | 0.007 | 0.01 | -0.009; 0.04 | 0.004 |
| *c*‘ | 0.07 | 0.07 | -0.07; 0.21 | 0.05 | -0.08 | 0.08 | -0.24; 0.09 | -0.04 |
| *c* | 0.09 | 0.07 | -0.05; 0.22 | 0.07 | -0.07 | 0.08 | -0.23; 0.10 | -0.04 |
|  |  |  |  |  |  |  |  |  |

*Unstandardized Regression Coefficients, Bootstrap Confidence Intervals, and Model Information for the Mediation Model, Controlling for Potential Confounders*

***Note***. The analyses controlled for study condition, ADHD and ODD baseline symptoms, baseline parenting behaviour, child age and child sex. The ADHD model additionally included post-assessment ODD symptoms as covariate; the ODD model additionally included post-assessment ADHD symptoms as covariate. Sample size *n* = 232. *a*_1_: parental internalizing symptoms (i.e., symptoms of depression, anxiety, and stress) at baseline 🡪 negative parenting behavior at three-month interim assessment, *b*_1_: negative parenting behavior at three-month interim assessment 🡪 outcome at post-assessment, *a*_1_*b*_1_: indirect effect of parental internalizing symptoms on outcome through negative parenting behavior, *c‘*: direct effect of parental internalizing symptoms on outcome, *c*: total effect of parental internalizing symptoms on outcome, ADHD = attention-deficit/hyperactivity disorder, ODD = oppositional defiant disorder, Coeff. = unstandardized regression coefficient, SE = standard error, CI = confidence interval. * significant coefficient (95% CI). The standard errors and confidence intervals for the total effects were determined without the use of bootstrap samples. Study condition was included as a confounding variable in both models.
